# Optimization of Process Parameters for the Laser Polishing of Hardened Tool Steel

**DOI:** 10.3390/ma15217746

**Published:** 2022-11-03

**Authors:** Bastian Meylan, Ivan Calderon, Kilian Wasmer

**Affiliations:** 1 Swiss Federal Laboratories for Materials Science and Technology (Empa), Laboratory of Advanced Materials Processing (LAMP), CH-3602 Thun, Switzerland; 2 Unitechnologies S.A., CH-3238 Gals, Switzerland

**Keywords:** laser polishing, tool steel, surface roughness, design of experiments, residual austenite

## Abstract

In mold making, the mold surface roughness directly affects the surface roughness of the produced part. To achieve surface roughness below 0.8 μm, the cost of surface finish is high and time-consuming. One alternative to the different grinding and polishing steps is laser polishing (LP). This study investigates and models the LP of tool steel (X38CrMoV5-1-DIN 1.2343), typical for the mold industry, having an initial rough surface obtained by electrical discharge machining. The microstructures of the re-melted layer and heat-affected zone due to the LP process were also studied. Four parameters: the laser spot size, velocity, maximum melt pool temperature and overlapping were investigated via a design of experiments (DoE) approach, specifically a factorial design. The responses were line roughness (*Ra*), surface roughness (*Sa*), and waviness (*Wa*). The surface topography was measured before and after the LP process by white light profilometer or confocal microscopy. DoE results showed that the selected factors interact in a complex manner, including the interactions, and depend on the responses. The DoE analysis of the results revealed that the roughness is mainly affected by the velocity, temperature and overlap. Based on a first DoE model, an optimization of the parameters was performed and allowed to find optimum parameters for the LP of the rough samples. The optimum conditions to minimize the roughness are a spot size of 0.9 mm, a velocity of 50 mm/s, a temperature of 2080 °C and an overlap of 90%. By using these parameters, the roughness could be reduced by a factor of almost 8 from 3.8 µm to approximately 0.5 µm. Observations of the microstructure reveal that the re-melted layer consists of columnar grains of residual austenite. This can be explained by the carbon intake of the electro-machined surface that helps stabilize the austenitic phase.

## 1. Introduction

In mold making, the mold surface roughness is a very important parameter. The reason is that the surface finish of the molding area directly influences the surface roughness of the produced part. In many cases, the process to achieve the final surface roughness is made in two steps. First, an initial roughness is obtained via electric discharge machining. It is often followed by either a grinding or a polishing step. The standard processes are contact methods using either an abrasive material or applying a chemical treatment and so are demanding in terms of raw material and chemicals. However, hard materials, such as hardened steel, are difficult to polish with these methods, including mechanically [1,2]. Consequently, for such hard material, to achieve surface roughness below 0.8 μm, the cost of surface finish is not only high but also time-consuming. One promising alternative to the grinding and polishing processes is the laser polishing (LP) process. On the opposite to the standard processes, the LP process is particularly interesting as it is not only a contactless method subjected to no or very little wear of materials but, in addition, is also energy efficient.

LP is a process where laser irradiation is employed to reduce the surface roughness of materials without any mechanical contact either by means of melting [3] or ablating [4] a thin layer of the surface. LP of metals is reported since the mid-2000s [5,6] and is considered by many industries as a conceivable alternative to the expensive and time-consuming standard polishing methods [2]. In recent times, LP has gained new interest as a way to improve the surface quality of samples produced by additive manufacturing (AM) [7,8,9,10,11,12,13,14]. Manco et al. [7] and Annamaria et al. [8] wrote two detailed reviews of this process for AM samples. Moreover, the LP process was investigated on the most common materials printed using an AM process; Obeidi et al. [9] on 316 L stainless, Li et al. [10] on Ti6Al4V, Yung et al. [11] on CoCr, and Richter et al. [12] on Co-Cr-Mo Alloy, and Dadbakhsh et al. [13] on Inconel 718. In contrast, tool steels such as H11 [15] or X38CrMoV5-1 [16] are much less investigated.

The principle of LP of metals is to melt a small volume of the material by a laser beam also known as the conduction regime. During the melting, the roughness is flattened due to the surface tension aided by increased mobility of the liquid state [2,3,7,8,9,11,17]. In addition, the LP process of metals can be divided into two categories that are related to the type of laser source; which are continuous wave (CW) or pulsed lasers [18]. In general, CW laser produces a large melt pool (10–80 µm deep) and so is employed for samples having significant roughness and it is referred to as macro-polishing. In contrast, the pulsed laser creates a very small melt pool (<5 µm deep) during each pulse and is utilized for samples with smaller roughness, and it is therefore referred to as micro-polishing [7,8,17,19]. In micro-polishing, the liquid state lasts, normally, shorter than the pulse repetition rate. In other words, the melt pool is not continuously present during the LP process [17].

Despite the promises of this technology, the LP process is still not widespread in industrial applications. The major argument lies in the difficulty to polish perfectly a metal surface with this technology. This was pointed out by Nüsser et al. [17], who disclosed that the LP process itself produces some kind of surface structures. These structures necessitate antagonist actions to avoid their occurrence. In other words, it is impossible to generate a surface without any structures or roughness with a single track of LP. A straightforward solution to overcome this obstacle is to perform several LP steps or combine various LP processes. For example, it is possible to start by having a step of macro-polishing followed by a step of micro-polishing. This approach was successfully demonstrated by Temmler et al. [18].

The Design of Experiment (DoE) approach is often employed for optimizing process parameters and was already applied for LP but mainly for AM processes [9,13,14]. The reason is that the main difficulty in applying DoE to laser processing is to set meaningful values for the power of the laser. Indeed, for example, for a fix laser power, variations of the velocity will lead to either heat, melt or even vaporize the material. In many studies, the energy density (Power/(Spot size·Velocity)) is usually chosen as the power-related parameter influencing the LP process [2,13,14,20]. In other contributions, the investigated parameters are related to the laser characteristic, in particular, the laser spot size, power and velocity [13,14], and a few on additional parameters such as laser scan passes, beam focal position, and percentage overlap of the laser tracks between consecutive passes [9].

The main novelty of this work is in the choice of the selected factors. For the first time, the numerically simulated maximum temperature of the melt pool as the power-related parameters were chosen. This has the advantage of allowing direct comparison of the different experiments realized at different speeds or with different spot sizes. The inconvenience is, of course, the need to solve the numerical models to obtain the laser power parameters which is more time-consuming and not as easily calculated as the power density. The supplementary parameters selected in the DoE are the sample velocity, the laser spot size and the overlap between two lines (related to the hatching distance).

## 2. Experimental Details

### 2.1. Design of Experiment

**Figure 1:**
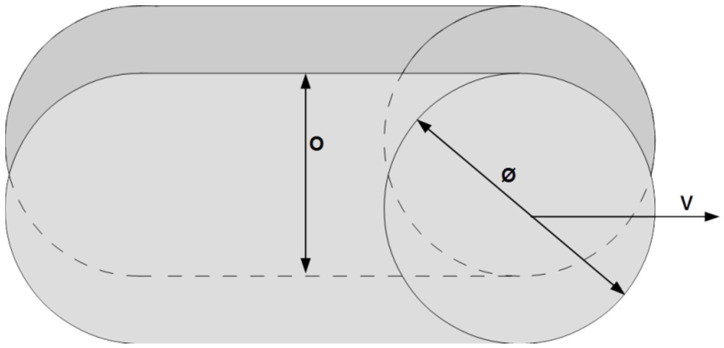
Schematic illustration of the DoE parameters studied. *Ø* is the laser spot diameter; *ν* the velocity of the displacement; *O* is the % overlap between two successive lines. The last parameter is the maximum temperature *T*, related to the laser power of the source.

It is well known that the parameter space in laser processes is very large and can be divided into three main categories: laser beam characteristics, process parameters, and material properties. Each of these categories encompasses at least six parameters, which makes testing every single parameter impossible. An interesting approach to overcome such obstacles is the use of Design of Experiment (DoE). In particular, factorial design allows analyzing simultaneously numerous factors and the estimation of both their individual and interaction effects. First, we employed a two-level factorial design in order to find the main parameters and/or interactions influencing the polishing and to find the direction of the optimum [21]. For factor screening experiments, such as laser processing, *two-level factorial design* or *2^k^* factorial design, where *k* is the number of factors and each factor can have 2 levels, is often employed [21]. As mentioned in the introduction, laser power and velocity are the main parameters taken into account in most studies on LP. In contrast, in this study and based on previous experimental works on laser processing [16,22,23,24], we selected four different factors, and they are: the spot size (*Ø*), the velocity (*ν*), the maximum temperature (*T*) and the overlap (*O*) between two successive parallel lines as illustrated in Figure 1. The limits of the values were set after some preliminary tests to insure a complete line of fusion on the sample [16]. Each factor is depicted by a low and a high level where their real values and the corresponding coded values are listed in Table 1. The experimental matrix is achieved by mixing the low and the high-level values of Table 1, and it is given in Table 2. This design allows determining the effect of the main parameters and their interactions in 16 experiments. Equation (1) shows our first-degree polynomial model with interactions:

(1) $$Y=a_{o}+\overset{4}{\underset{i=1}{\sum }}a_{i}X_{i}+\overset{4}{\underset{i\neq j}{\sum }}a_{ij}X_{i}X_{j}+\overset{4}{\underset{i\neq j\neq k}{\sum }}a_{ijk}X_{i}X_{j}X_{k}+a_{1234}X_{1}X_{2}X_{3}X_{4}+\varepsilon$$

where *Y* is the experimental response, *a*_0_ is a constant, *X_i_* is the factor, *a_i_* is the main effect coefficient (half-effect) associated to the factor *X_i_*, *a_ij_* is the 2 factors interaction effect coefficients (half-effect), *a_ijk_* is the 3 factors interaction effect coefficients (half-effect), *a*_1234_ is the 4 factors interaction effect coefficients (half-effect) and, finally, *ε* is the error in the response *Y* (also known as the residual).

Although perfect linearity in the factor effects is unneeded, this hypothesis is frequently a latent concern when using a *two-level factorial design* [21]. Therefore, we decided to add a center point, often referred to as a zero level, in order to determine whether or not a curvature exists in the DoE model. As we only had two optics at disposition for this study, we could not test an intermediate spot size. Hence, for each spot size, two center points were made as shown at the bottom of Table 2.

In terms of responses, we selected the three most common measures in topography: they are the line roughness (*Ra*), surface roughness (*Sa*), and waviness (*Wa*). These parameters will be defined in Section 2.3 and their experimental measurements are given in Table 2.

**Table 1:** Factors, levels and coded values of the design of experiments.

Factors	Symbol	Unit	Low Level	Center Point	High Level
Spot size	*Ø*	[mm]	0.6		0.9
Velocity	*v*	[mm/s]	50	100	150
Temperature	*T*	[°C]	1660	1800	1940
Overlap	*O*	[%]	60	75	90
**Code**			**−1**	**0**	**1**

 

**Table 2:** Table of experiments including the coded values (first column) of the main parameters and the actual values (columns 2–5), other laser parameters calculated from the main parameters (column 6–8) and the responses issued from topography measurements (last 3 columns).

Tests Coded Factors	DoE Parameters Real Values				Other Laser Parameters			Responses		
	*Ø*	*V*	*T*	*O*	Power	Hatching	Lines	*Ra*	*Sa*	*Wa*
	[mm]	[mm/s]	[°C]	[%]	[W]	[µm]		[µm]	[µm]	[µm]
DoE 1 (−1, −1, −1, −1)	0.6	50	1660	60	192	240	33	1.29	2.41	1.64
DoE 2 (−1, −1, −1, 1)	0.6	50	1660	90	192	60	133	1.02	1.94	1.24
DoE 3 (−1, −1, 1, −1)	0.6	50	1940	60	240	240	33	1.08	1.89	1.07
DoE 4 (−1, −1, 1, 1)	0.6	50	1940	90	240	60	133	0.74	1.55	1.07
DoE 5 (−1, 1, −1, −1)	0.6	150	1660	60	263	240	33	1.90	2.82	1.34
DoE 6 (−1, 1, −1, 1)	0.6	150	1660	90	263	60	133	1.50	2.43	1.33
DoE 7 (−1, 1, 1, −1)	0.6	150	1940	60	328	240	33	1.61	2.22	1.13
DoE 8 (−1, 1, 1, 1)	0.6	150	1940	90	328	60	133	1.15	2.27	1.44
DoE 9 (1, −1, −1, −1)	0.9	50	1660	60	320	360	22	1.25	2.18	1.21
DoE 10 (1, −1, −1, 1)	0.9	50	1660	90	320	90	89	1.13	2.31	1.56
DoE 11 (1, −1, 1, −1)	0.9	50	1940	60	399	360	22	1.03	1.85	1.08
DoE 12 (1, −1, 1, 1)	0.9	50	1940	90	399	90	89	0.77	1.50	0.98
DoE 13 (1, 1, −1, −1)	0.9	150	1660	60	457	360	22	2.10	2.89	1.21
DoE 14 (1, 1, −1, 1)	0.9	150	1660	90	457	90	89	1.77	2.56	1.20
DoE 15 (1, 1, 1, −1)	0.9	150	1940	60	572	360	22	1.22	2.07	1.12
DoE 16 (1, 1, 1, 1)	0.9	150	1940	90	572	90	89	1.11	2.07	1.22
Center 1 (−1, 0, 0, 0)	0.6	100	1800	75	260	150	53	1.16	2.22	1.56
Center 2 (−1, 0, 0, 0)	0.6	100	1800	75	260	150	53	1.21	2.10	1.27
Center 3 (1, 0, 0, 0)	0.9	100	1800	75	444	225	36	1.12	1.87	1.02
Center 4 (1, 0, 0, 0)	0.9	100	1800	75	444	225	36	1.41	2.29	1.27

 

After the first round of the DoE, the direction of the optimum was found (see Section 3—Results and Discussion). This means that a second DoE was performed with the significant parameters varied in the region of the optimum. The optimization parameters are shown in Table 3. In this case, only the velocity and the temperature are varied whereas the spot size and overlap are kept at the optimum found after the first DoE. The table of experiments for the optimization is shown in Table 4.

**Table 3:** Factors, levels and coded values of the design of experiments.

Factors	Symbol	Unit	Low Level	Center Point	High Level
Spot size	*Ø*	[mm]			0.9
Velocity	*v*	[mm/s]	20	35	50
Temperature	*T*	[°C]	1940	2080	2220
Overlap	*O*	[%]			90
**Code**			**−1**	**0**	**1**

 

**Table 4:** Matrix of experiments for the DoE optimization including the coded values (first column) of the main parameters and the actual values (columns 2–5), other laser parameters calculated from the main parameters (column 6–8) and the responses issued from topography measurements (last 3 columns).

Tests Coded Factors	DoE Parameters Real Values				Other Laser Parameters			Responses		
	*Ø*	*v*	*T*	*O*	Power	Hatching	Lines	*Ra*	*Sa*	*Wa*
	[mm]	[mm/s]	[°C]	[%]	[W]	[µm]		[µm]	[µm]	[µm]
Optim 1 (1,−1,−1,1)	0.9	20	1940	90	326	90	89	0.82	1.72	1.17
Optim 2 (1,−1,0,1)	0.9	20	2080	90	359	90	89	0.56	1.61	1.19
Optim 3 (1,−1,1,1)	0.9	20	2220	90	391	90	89	0.55	1.74	1.19
Optim 4 (1,0,−1,1)	0.9	35	1940	90	365	90	89	0.72	1.62	1.05
Optim 5 (1,0, 0,1)	0.9	35	2080	90	401	90	89	0.57	1.32	0.89
Optim 6 (1,0,1,1)	0.9	35	2220	90	438	90	89	0.52	1.32	0.99
Optim 7 (1,1,−1,1) = DoE 12	0.9	50	1940	90	399	90	89	0.76	1.50	0.98
Optim 8 (1,1,0,1)	0.9	50	2080	90	438	90	89	0.58	1.34	0.89
Optim 9 (1,1,1,1)	0.9	50	2220	90	478	90	89	0.53	1.57	1.30

### 2.2. Thermal Modeling in the Perspective of Tool Steel Laser Polishing

In Section 2.1 and Figure 1, we defined four factors that are investigated in this study. One of the factors considered is the maximum temperature (*T*). The major advantage of using this parameter is that it allows direct comparison of the different experiments realized at different speeds or with different spot sizes. In this work, the maximum temperature has been determined via numerical simulations developed in a prior work by Meylan et al. [22]. Hence, only a summary of the model/methods is presented in this work.

**Figure 2:**
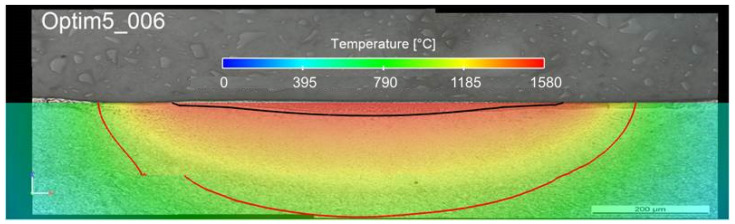
Comparison between FEM simulation based on the model developed by Meylan et al. [22] and an experiment using the process parameters of Optim5 in Table 4.

To identify the melt pool created by a laser, finite element simulation software was used by Meylan et al. [22] to model the heat transfer in a 3D part that has a shape in the form of a parallelepiped. In this contribution, the main problem was to retrieve the two space- and time-dependent fields. They are the temperature *T* (unit °C) and the specific enthalpy *u* (unit *J/g*). To address this issue, the temperature was considered dependent on the local specific enthalpy which includes the majority of the thermal properties of the material and in particular the heat capacity *C_p_*, latent heat of fusion *L*, and melting temperature *T_f_*. Then, the fields of the specific enthalpy and temperature had to fulfill the equation of the heat diffusion (energy conservation) for all time steps. In terms of initial and boundary conditions, we imposed an ambient initial condition *u* = *u*_0_ at time *t* = 0. In addition, on the six faces of the parallelepipedon, we also imposed some boundary conditions for the temperature even though only the boundary conditions on the surface processed by the laser (*z* = *H*) are pertinent if the domain is sufficiently large when compared to the polished region. Finally, Meylan et al. [22] used a space discretization that was gotten by applying a standard finite element method (FEM) (Galerkin formulation) in agreement with Kolossov et al. [25]. The material properties of non-linearity are implemented via a semi-implicit method. Nevertheless, to obtain a better representation of the distribution of the laser intensity at a sub-millimetric scale while keeping the number of nodes required to mesh the entire domain acceptable, we employed a local refinements method developed by Boillat [26]. The thermal model investigated the variation of two distinct absorption coefficients that is when the material is solid (*a_s_*) and liquid (*a_l_*). To achieve this goal, a sensitivity analysis of both coefficients was carried out for values ranging between 0.20 and 0.35. This thermal model was validated via LP experiments using an austenitization temperature equal or higher than 950 °C for the heat-affected zone (HAZ) and a melting temperature of 1505 °C and higher. In terms of results, the best ones were achieved using two separate constants for the absorption coefficients. The first one was for the solid (*a_s_* = 35%) and it was based on the measurement at room temperature. The second was for the liquid (*a_l_* = 25%) and it was fixed so that the model fits the experimental data. An example of such simulation for Optim 5 is shown in Figure 2. All details on the thermal model can be found in Meylan et al. [22].

### 2.3. Laser Setup, Materials and Characterization Methods

**Figure 3:**
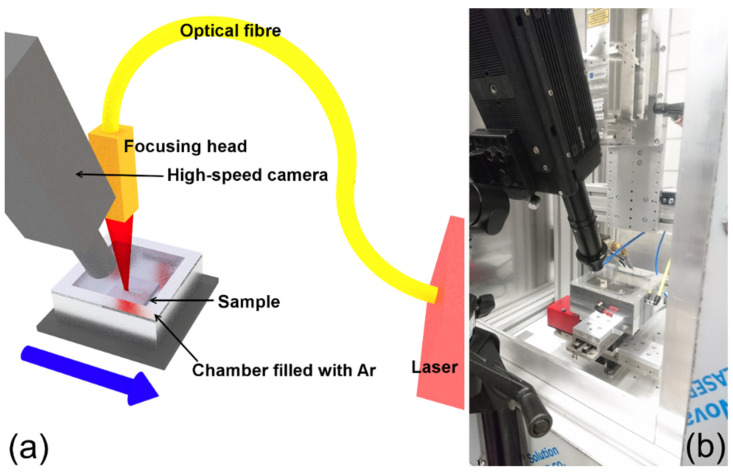
(**a**) Schematic representation of the experimental setup of LP. (**b**) photography of the actual setup. Taken from Meylan et al. [22]; published by MPDI under CC BY 4.0.

A continuous wave (CW), high power diode laser LDM 1000 from Laserline GmbH with a 980 nm wavelength was employed in this work. Two focusing heads were selected to provide two spot sizes selected in the DoE. The first one had a working distance of 297 mm with a focused spot size of 0.9 mm at the focal point (1/*e*^2^). The second, with a working distance of 188 mm, provides a focused spot size of 0.6 mm at the focal point (1/*e*^2^). In terms of laser beam shape, we chose a flat top beam to guarantee that there is no overheating at the center of the laser beam. Moreover, as the laser head was fixed, we mounted the sample on an *x*-*y* table allowing movement in both directions. To circumvent oxidation during the LP process, the sample and table were installed in a chamber that was filled with argon. Figure 3 shows a schematic drawing and an image of the full setup. 

To avoid overheating at the beginning and end of each line, the movement of the sample started first in the *x*-direction. Once the sample reached a constant speed, the laser was triggered. Third, the laser remained switched on for a displacement of 19 mm. After the laser was stopped, the table was decelerated to a stop. Next, the table was moved by the hatching distance in the *y*-direction as defined in Table 2 and Table 4. Finally, the LP process could begin once again but in the reverse direction along the *x*-axis. This procedure was repeated by the number of lines (also shown in Table 2 and Table 4) in order to ensure coverage of an area of 19 × 8 mm^2^.

 

In this investigation, the LP process was carried out on two plates of the size of 70 × 70 × 7 mm^3^ made of X38CrMoV5-1 (DIN 1.2343) tool steel. On the top surface, an area of 50 × 50 mm^2^ was produced by electric discharge machining (EDM). The final surface state was CH30 on the Charmilles scale for EDM and this corresponds to a line roughness *Ra* of 3.15 µm.

The topography of the surfaces before and after the LP process was measured with a withe light profilometer Altisurf 500 from Altimet, equipped with an optical pen OP300WM from Stil SA. The given specifications are a maximum vertical range of 300 µm and an axial resolution of 10 nm. Surfaces of 17 × 7 mm^2^ were scanned with a lateral step of 30 µm. The region observed was taken at the center of each LP surface of 19 × 8 mm^2^ to avoid taking into account the edges of the LP surfaces. The line roughnesses (*Ra*) were first calculated on each surface according to the norm ISO 25178 [27] and are reported in Table 2 and Table 4. After, a robust Gaussian filter was applied on the surface with a cut-off value of 0.8 mm to separate the surface roughness (*Sa*—wavelength lower than 0.8 mm) and the waviness (*Wa*—wavelength larger than 0.8 mm) of the surface. The *Sa* values were also calculated according to the norm ISO 25178 [27] on one of the two filtered surfaces and are reported in Table 2 and Table 4. Similarly, the *Wa* values are given in the same tables for the second of the two filtered surfaces.

The re-solidified area and heat-affected zone produced by the LP were observed on cross-sections of single-line experiments produced with the same laser parameters as for the DoE optimization (Table 4) but with no overlapping. Hence, the first step was to cut the plates with an Accutom 5 from Struers. Afterward, the cross-sections were ground and polished up to colloidal suspension until a mirror-polished surface is reached. After, they were etched for 20 s with a 5% Nital solution and examined with an Axioplan optical microscope from Zeiss. The cross-sections were then re-polished, and ion milled for Electron Backscatter Diffraction (EBSD) observations. The EDSD maps were made using two models of the scanning electron microscope (SEM). The first one was a tungsten filament DSM 962 SEM from Zeiss, and the second, a field emission Lyra 3 SEM from TESCAN with EBSD DigiView 5 camera and EDAX OIM software version 7.0.

## 3. Results and Discussion

### 3.1. First DoE Model Based on the Line Roughness (Ra)

**Figure 4:**
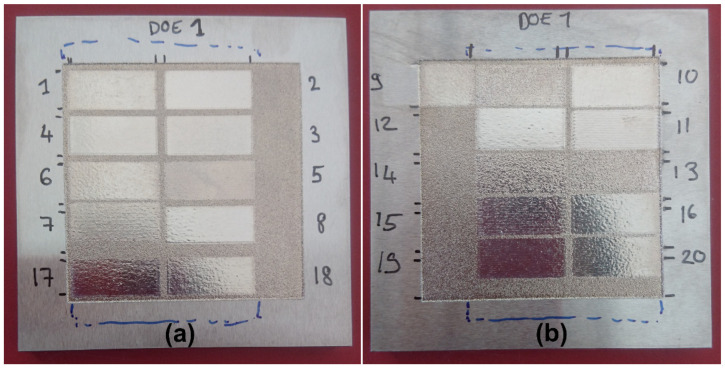
Pictures of the DoE surfaces after LP for a spot size of (**a**) 0.6 mm and (**b**) 0.9 mm.

The DoE surfaces are presented in Figure 4 and the topography measurements are given in Table 2. As can be seen from the figure and table, the surfaces have very different aspects and also topographical values. The experiments at high velocity and low temperature are very much at the limit of melting the material. This may come from the approximations in the model and also some uncertainties on the parameters used in the simulations, especially the absorption coefficient. This is not a problem for these first experiments as they are basically a screening of the parameters in order to find their influence for later optimization.

**Figure 5:**
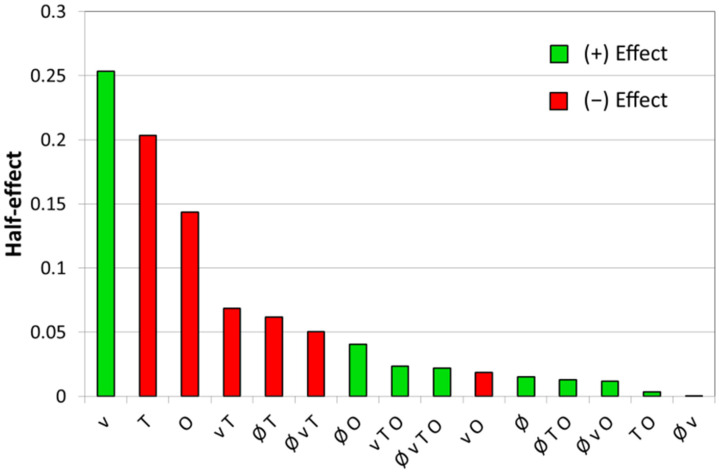
Bar charts showing the half-effects of the parameters and interactions in decreasing order. A positive effect means an increase in the parameter leads to an increase in the roughness, a negative effect does the opposite.

A proper statistical analysis of the DoE values obtained for the line roughness *Ra* revealed the effects of the parameters and interactions and they are given in Figure 5 and Equation (3). This analysis of the parameters shows that three main parameters; the velocity (*v* = +0.25), temperature (*T* = −0.20) and overlap (*O* = −0.14) have the biggest influence on the line roughness *Ra*. In contrast, all interactions as well as the spot diameter have a much lesser influence. Thus, for the DoE model, only these three main influencing parameters were kept. To confirm that these parameters are relevant and significant, an *ANOVA* with a 95% confidence interval was performed and the results are shown in Table 5. In this table, only the terms with a *p*-value (probability of error) equal or less than 0.05, which gives a 95% confidence interval or higher, are considered significant. In contrast, terms with *p*-values larger than 0.1 are neglected. The *F* value presented in Table 5 is defined as:

(2) $$F=\frac{MS_{Terms}}{MS_{Residual}}$$

where the mean square of terms (*MS_Terms_*) is the ratio of the sum of squares within terms to its degree of freedom $\left(SS_{Terms}/DF_{Term}\right)$, and similarly $MS_{Residual}=SS_{Residual}/DF_{Residual}$. Equation (2) is a statistical test to verify the hypothesis that no differences exist between the two means (also known as a “null hypothesis”). If so, it means that the observed difference is due to either chance or noise alone. It is common practice that for effects with *F* values larger than three times their standard error (residual) (*F* > 3), the null hypothesis is rejected [28]. Inspection of Table 5 indicates that the model constructed from the selected main factors has an *F* value of about 14.5 indicating that the model is significant. From this table, the model has a *p*-value of 0.0001 revealing that there is only a 0.01% probability that this model occurs due to noise. The fact that all *p*-values are less than 0.1 confirms that they all are significant so the model achieved a 95% certainty.

 

**Table 5:** Analysis of variance (*ANOVA*) for *Ra* (*SS*: Sum of square; *DF*: degree of freedom; *MS*: mean square).

Parameter	*SS*	*DF*	*MS*	*F*	*p*-Value
*Model*	1.72	3	0.57	14.51	0.0001
*V*	0.87	1	0.87	21.94	0.0003
*T*	0.35	1	0.35	8.94	0.0092
*O*	0.24	1	0.24	6.19	0.0251
Curvature	7.15 × 10^−3^	1	7.15 × 10^−3^	0.18	0.6765
Residual	0.59	15	0.039		
Lack of Fit	0.16	12	0.013	0.094	0.9989
Pure Error	0.43	3	0.14		
Total	2.32	19			

As mentioned in Section 2.1, although the model is assumed to be linear, the *ANOVA* proves that the *2^k^* factorial design functions very well [21]. Nevertheless, if the model was built taking into account the interaction terms, the model may be subjected to quadratic effects or second-order curvature [21]. Under such circumstances, it is advised to control the model for curvature, and this can be achieved by adding center points. In Table 5, the *p*-value for the curvature is 0.68, which indicates that the quadratic effects seem not significant and so we can neglect the curvature assumption. We can conclude that this first-order model with the main factors and interactions is suitable.

The final equation in terms of coded values is written as follows:

(3) Ra=1.28+0.25 v−0.2 T−0.14 O

**Figure 6:**
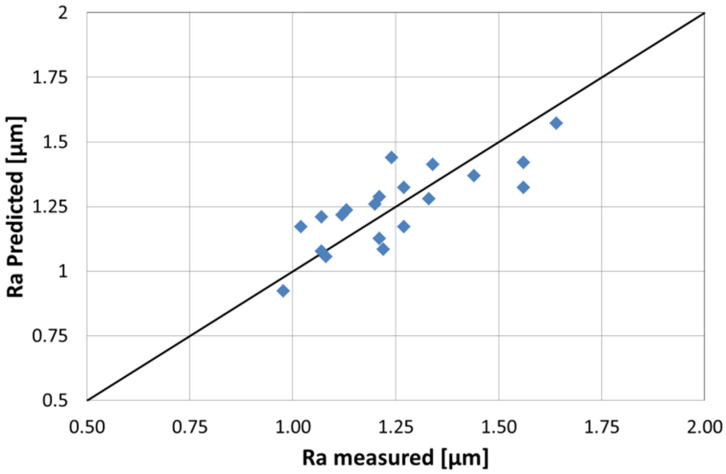
Predicted *Ra* vs. measured *Ra* for the first DoE model.

The *R*-square value (*R^2^*), adjusted-*R^2^* and predicted-*R^2^* of this model are 0.91, 0.86, and 0.79, respectively. The *R^2^* is high enough for a simple semi-empirical model with just three main effects to describe the 16 experiments. The adjusted-*R^2^* for this simple model is very close as only three terms are kept in this model. A large difference between *R^2^* and adjusted-*R^2^* could be a sign of overfitting which is not the case for this model. The predicted-*R^2^* is also good which indicates that the model correctly predicts the measured point as illustrated in Figure 6 which plots the predicted-*Ra* (calculated from Equation (3)) versus the experimentally measured values. A high correlation coefficient of 0.93 is observed.

 

 

Based on the above analysis, we also found that the velocity is the main effect influencing the line roughness *Ra*. One of the explanations for this result could be that an increase in the laser velocity tends to create a more elongated melt pool as observed via the FEM simulation in Meylan et al. [22] (i.e., the width perpendicular to the travel direction becomes shorter). A narrower melt pool means a redistribution of the material on a smaller surface and so a reduced efficiency of the LP process. Another effect of a narrow melt pool is a larger temperature gradient that can lead to an increase in thermos-capillary flows and so be detrimental to the LP process. Finally, a lower velocity also implies more time for the redistribution of the material in the liquid state. As suggested by the model, a decrease in the velocity below the values tested in the first DoE may still have the potential to improve the roughness and, thus, we decided to vary this parameter to lower velocities for the DoE optimization. 

The next parameter having an influence on *Ra* is the temperature. An increase in the temperature, and, by consequence, the laser power, also produces a larger and deeper melt pool. This means, as for the velocity, a redistribution of the material over a larger surface with the possibility of removing larger fluctuations of the surface. It is particularly true for rough samples such as the one employed in this study where the peak-to-valley height difference can be over 30 µm. Hence, it is necessary to produce a deep enough melt pool to eliminate this kind of surface variation. As for the velocity, the temperature will be varied in the DoE optimization as the model shows the potential for more roughness reduction at a higher temperature.

 

The overlap is the last main factor to have a significant influence on *Ra*. In LP, a large overlap is also beneficial, and this was also observed by Chow et al. [29]. A large overlap means that a large part of the previous line is re-melted and re-processed. This can improve the LP as it can remove some surface structure produced by the LP, in particular at the edge of the melted zone. Our model predicts also a better roughness for higher overlap. However, an increase in the overlap also leads to an exponential increase in the number of lines and so of processing time. In order to keep the process profitable, the overlap was kept to 90% for the DoE optimization. 

Finally, the spot size diameters tested in this study do not have a significant influence on the line roughness *Ra*. By definition, the line roughness is a measure of the surface variations occurring over short lateral distances (in our case, the filter was set at 0.8 mm). It would still be expected that a smaller spot size of 0.6 mm does not remove the defects with a wavelength between 0.6 and 0.8 mm. However, it seems not to be the case as no significant differences are observed between the two tested spot sizes. A reason can be due to the Gaussian filter with a cut-off set at 0.8 mm. Actually, a Gaussian filter does not go from 100% transmission to 0% as a step function but gradually and so the filtering certainly starts to already cut some wavelengths above 0.5 mm. This would mean that the influence of the wavelength between 0.6 and 0.8 mm is reduced as compared to the shorter wavelength on the measurement of the *Ra*. 

**Figure 7:**
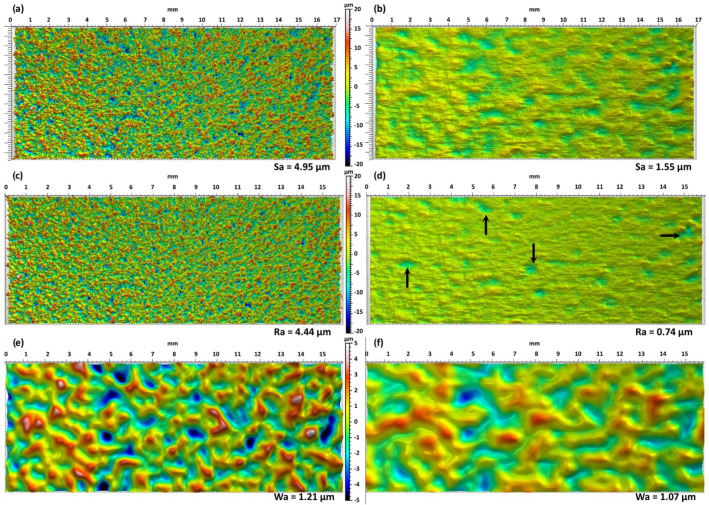
Topography measurement for the test DoE4 before (left-hand side) and after (right-hand side) LP. (**a**,**b**) show the unfiltered filtered (*Sa*), (**c**,**d**) show only the roughness of the surface with a cut-off of 0.8 mm (*Ra*) (**e**,**f**) show only the waviness of the surface with a cut-off of 0.8 mm (*Ra*). For each line, the vertical color scale is the same in order to compare the surfaces and is indicated in the middle. The scale for the waviness is lower as the original waviness is much lower than the original roughness (1.21 µm vs. 4.44 µm).

Figure 7 shows a typical example of a 17 × 7 mm^2^ surface before and after LP with the conditions of DoE 4 in Table 2. Figure 7a,b are the unfiltered surfaces that are a combination of roughness and waviness. Figure 7c,d show only the roughness after filtering and a cut-off of 0.8 mm. Finally, Figure 7e,f are only the waviness part of the surface (beware of the change of the height scale for the waviness). By comparing the images before and after the LP process, a big reduction in the *Sa* value is observed and a detailed inspection shows that most of the reduction is due to the diminution of the roughness (Figure 7c,d). Indeed, the roughness map after LP (Figure 7d) is mostly flat (single color yellow-green) with just a few shallow craters (see black arrows) randomly distributed over the surface. In contrast, the waviness map is only slightly affected by the LP process. The highest pics (in white) and lowest valleys (dark blue) have disappeared, and this explains the small reduction in the *Wa* value from 1.21 to 1.07 µm. This result is not surprising since the waviness maps represent surface defects with a wavelength over 0.8 mm and the spot sizes used in the study were 0.6 and 0.9 mm with melt pool size slightly below these values. Therefore, the LP process cannot remove defects above a critical wavelength. Richter et al. [12] have recently adapted a criterion developed for pulsed micro-polishing from Perry et al. [30] to estimate the critical spatial frequency of defects that can be removed by LP. The width of the melt pool can be used as a first approximation for the critical wavelength (i.e., the inverse of the critical frequency) from the values presented by Richter et al. [12]. Hence, it is clear that LP has just a minimal influence on the LP of surface defects bigger than the melt pool size and it is confirmed by the present results.

### 3.2. First DoE Model Based on the Waviness (Wa)

**Figure 8:**
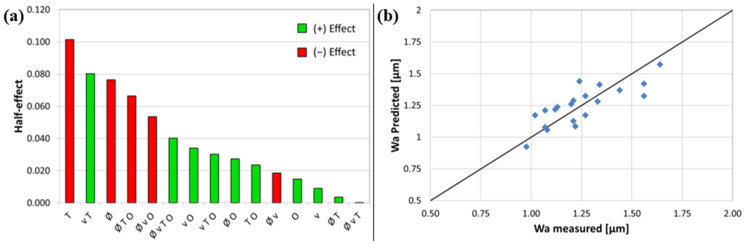
(**a**) Bar charts showing the half-effects of the parameters and interactions in decreasing order for *Wa*. A positive effect means an increase in the parameter leads to an increase in the waviness, a negative effect does the opposite. (**b**) *Wa* predicted with the waviness model versus actual measured values.

The same approach as for the roughness was applied to the waviness values shown in Table 2. The half-effects are shown in Figure 8a. For the waviness, the transition is not as clear between the significant parameters and the lesser ones. Consequently, as a first model, only the effects with values of the half-effect higher than |0.60| are selected. These effects are: *T*, *v*·*T*, *Ø*, and *Ø*·*T*·*O*. As can be seen, the model is not as simple as for the roughness as there are two interactions between the factors that are significant. In addition, it is found that some interactions contain main parameters that are themselves not significant. It is the case for the velocity and overlap. Even if it is common to consider all main parameters that are significant in the interactions, we decided to do otherwise. The main reasons were two-fold. First, we wanted to keep the model as simple as possible. Second, taking into account the insignificant main parameters decreased the reliability of the model. The *ANOVA* of the model with the selected effects is shown in Table 6. The spot size (*Ø*) parameter is just marginally significant as it is below 0.1, but above the 0.05 limit for 95% significance. This parameter was still kept as it improves the overall model. The curvature was not tested, as the spot size does not have a center point and is present in the model.

**Table 6:** Analysis of variance (*ANOVA*) for *Ra* (*SS*: Sum of square; *DF*: degree of freedom; *MS*: mean square).

Parameter	*SS*	*DF*	*MS*	*F*	*p*-Value
*Model*	0.43	4	0.11	7.2	0.0019
*Ø*	0.055	1	0.055	3.69	0.074
*T*	0.13	1	0.13	8.67	0.0101
*T·v*	0.13	1	0.13	8.52	0.0106
*Ø·T·O*	0.092	1	0.092	6.12	0.0258
Residual	0.22	15	0.015		
Lack of Fit	0.15	12	0.012	0.47	0.8471
Pure Error	0.077	3	0.026		
Total	0.65	19			

The final equation in terms of coded values is written as follows:

(4) Wa=1.26−0.091 T−0.054 ∅+0.09 Tv−0.077 ∅TO

The *R*-square value (*R^2^*), adjusted-*R^2^* and predicted-*R^2^* of this model are 0.66, 0.57, and 0.43, respectively. Although, these values are lower than the ones for *Ra*, this is not surprising since, as already explained, the LP process does not have a big influence on the waviness. This can be seen in the relatively low dispersion of the waviness values. As the process does not influence the waviness much, it is thus normal that the individual parameters have less effect on the waviness. The model is still acceptable as can be seen from the predicted value vs. the actual values plot in Figure 8b. A relatively high correlation coefficient of 0.79 is observed.

**Figure 9:**
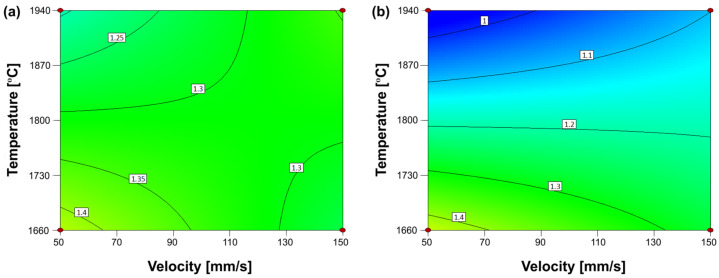
Process maps (speed vs. temperature) based on the first DoE waviness model according to Equation (4) for laser polishing for (**a**) small laser spot of 0.6 mm and an overlap of 90% and (**b**) a larger laser spot size of 0.9 mm and an overlap of 90%.

Based on this model, some process maps can be made. They revealed that the optimum corners of high temperature and low speed with a high overlap are profitable for the process. This is an excellent result as these observations are consistent with the results obtained for the roughness *Ra*. The main difference, in this case, is that the spot size has an influence as illustrated in Figure 9a,b. Both figures show the influence of the temperature and velocity for an overlap of 90%, but in the first case (Figure 9a) with the small spot size (0.6 mm) and the larger spot size (0.9 mm) in the second (Figure 9b). It is evident that the corner of high temperature and low velocity gives a lower waviness for the larger spot size. For this reason, the larger spot size was selected for the DoE optimization. A larger optic to test an even higher spot size was not possible to organize due to time and cost constraints.

### 3.3. Optimization of the DoE

**Figure 10:**
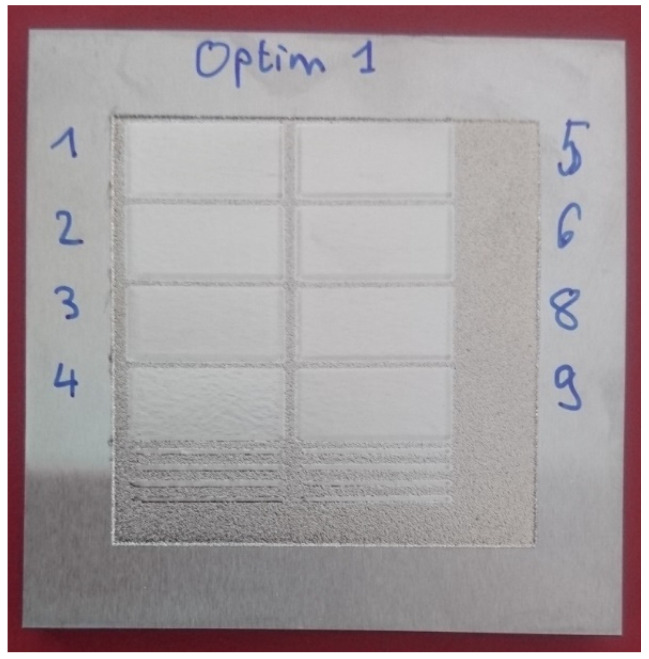
Pictures of the DoE optimization surfaces after LP with parameters as shown in Table 4 (Optim 7 is the same as DoE 12 and is not repeated here). Below the surfaces are also single lines with the same laser parameters without overlap.

**Figure 11:**
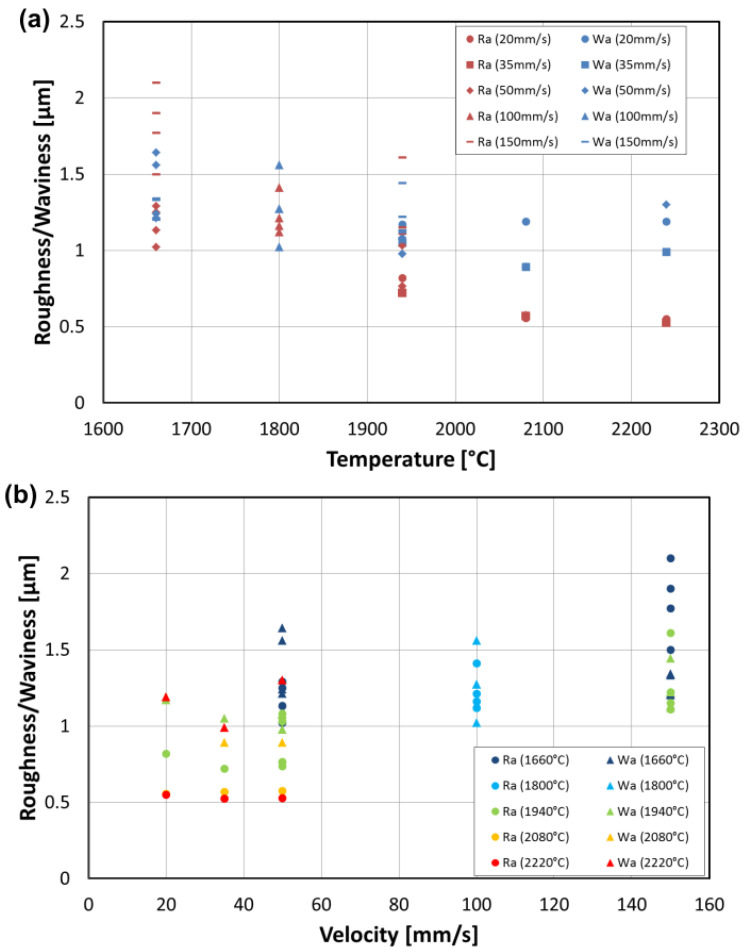
Evolution of the waviness and roughness as a function of (**a**) temperature and (**b**) velocity.

Following the results in Section 3.1 and Section 3.2, an optimization of the process parameters was performed to obtain the best LP with one scan. As explained, the temperature was increased, and the speed was lowered. In contrast, the overlap and spot size were kept constant at 90% and 0.9 mm to minimize the processing time. The test parameters and topography measurements are shown in Table 4. A picture of the surfaces obtained with new tests is also shown in Figure 10. Obviously, when comparing Figure 10 with Figure 4, all surfaces have been significantly improved with roughness well below 1 µm. The results are close to a minimum and so the linear assumption of the DoE is not valid. The evolution of the roughness and waviness as a function of temperature and velocity is shown in Figure 11a,b, respectively. Based on Figure 11a, an increase in the temperature to 2080 °C is still very beneficial for the LP process as it decreases both the roughness and waviness for a given velocity. Further increase in the temperature is not recommended as it does almost not affect the roughness and slightly increases the waviness of the surface. In Figure 11b, it is seen that reducing the velocity below 50 mm/s does not lead to a significant decrease in the roughness or waviness. Actually, at 20 mm/s, even an increase in the waviness is observed. From an industrial point of view, reducing the velocity increases the processing time and so increases the processing cost. Hence, it is not advised to decrease the velocity below 35 mm/s.

The results observed in our DoE optimization are consistent with several studies where an increase in the energy density (ED) from the melting point leads first to a decrease in the roughness to an optimal followed by an increase in the roughness [2,14,31]. Ukar et al. [2] attributed this transition from shallow surface melting (SSM) to surface over-melt (SOM). According to them, the transition occurs when the thickness of the melt pool increases above the peak-to-valley height, which creates a material melt pool. In this melt pool, they argue that the convection flow starts to be dominant and creates waves in the melt pool that augments the roughness of the re-solidified part. Based on our results, we believe that it is more likely that the convection effects are always present in the melt pool, but the optimum temperature marks a transition at which the reduction in the roughness by LP is outbalanced by the creation of roughness by LP through convective flow or Marangoni effect as both increases with the temperature.

**Figure 12:**
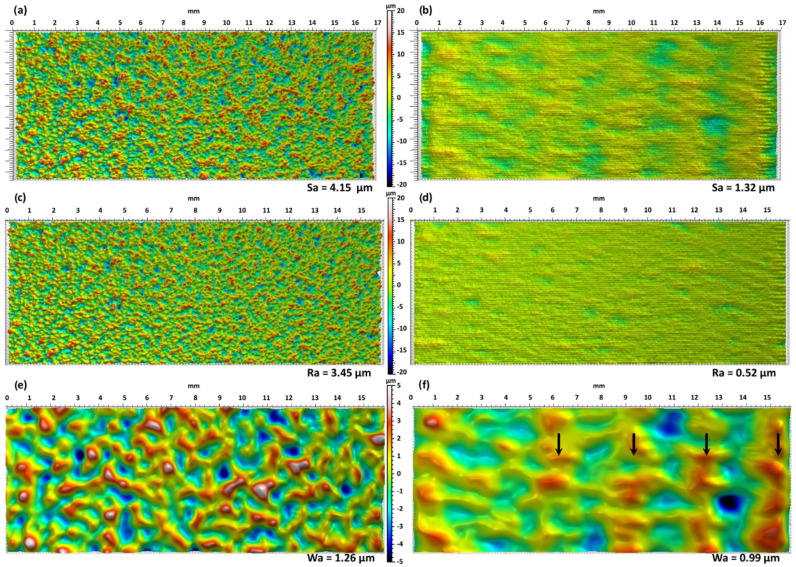
Topography measurement for the test Otpim 6 before (left-hand side) and after (right-hand side) LP. (**a**,**b**) show the unfiltered filtered (*Sa*), (**c**,**d**) show only the roughness of the surface with a cut-off of 0.8 mm (*Ra*) (**e**,**f**) show only the waviness of the surface with a cut-off of 0.8 mm (*Wa*). For each line, the vertical color scale is the same in order to compare the surfaces and is indicated in the middle.

A velocity decrease does not always lead to a better LP and the origin of this behavior is not perfectly clear. Preliminary results show that variations of the velocity due to the linear stage employed in this work could explain the periodic variations (see black arrows in Figure 12f) observed on the surface and so an increase in waviness. 

Similarly to Figure 7, Figure 12 shows the topography measurement but for the test Optim 6 (35 mm/s; 2220 °C). It can be observed that the roughness is one of the lowest in Table 4 and very close to the minimum possible with the current process. Already on the roughness measurement (Figure 12d), lines due to the process are visible and an increase in the temperature does not decrease these lines. The only way to make them disappear would be to make a second passage of LP or another polishing method. The waviness of the polished sample, in this case, has almost no common feature with the original surface (Figure 12e,f). The peaks and valleys in Figure 12e are not recognizable in Figure 12f. On the other hand, some periodic structures were added on the surface, and they might be due to the velocity variations of the linear stage. The major changes in the waviness go against the critical frequency limit developed for continuous laser [12] discussed previously. However, as noted by Richter et al. [12], the criterion already showed some errors especially to estimate the critical frequency in the direction of the displacement. The present results confirm the issue of directly transferring the critical frequency developed for pulse micro-polishing [30]. As a continuous melt pool is always present, the transport of material is, thus, possible over distances longer than the melt pool under certain conditions. The change of the waviness perpendicularly to the displacement is not easy to explain. It can be due to the high overlap used (90%) which means that each region of the sample saw 10 times the laser beam. This could, then, redistribute the material over and over again till the original waviness topography is lost.

### 3.4. Microstructure of the Re-Melted Layer and Heat Affected Zone

**Figure 13:**
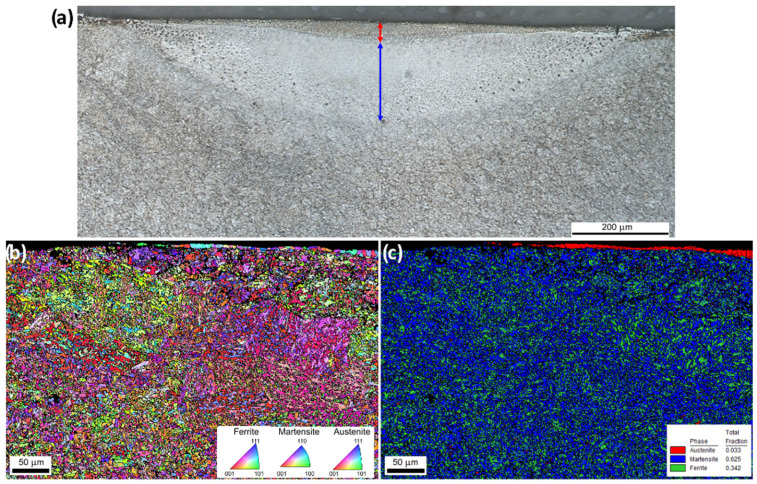
(**a**) optical micrograph after etching of the cross-section of a single line made with the laser parameter of Optim 9. (**b**) EBSD inverse pole figure (IPF) map in color-coded as well as the grain boundaries in black of the same cross-section. (**c**) EBSD phase map (color coded).

A typical microstructure of a single line of LP is shown in Figure 13a. The melted layer (see red arrow) has a maximum depth of 42 µm. Below the melted area, there is a large heat-affected zone (HAZ; see blue arrow). The HAZ appears clearly on the image as a lighter gray area with a maximum depth of around 210 µm. The HAZ consists mostly of untempered martensite and is almost not etched by the nital reagent. The round shapes visible at the periphery of the HAZ are, actually, not pores but an artefact resulting from the etching process. The bulk microstructure is found below the HAZ and consists of tempered martensite. The width and depth of the melted layer and HAZ are given in Table 7.

As expected, an increase in the temperature leads to an increase in the size of the melted layer and HAZ. A reduction in the velocity has the same effect. The depth of the melted layer is close to the depth-to-valley height (≈30–40 µm) for most of the tests apart from the ones at 20 mm/s and the two highest temperatures (≈60–90 µm). This is also a sign that these conditions are in the SOM regime [2] and explains the higher waviness obtained with these conditions.

**Figure 14:**
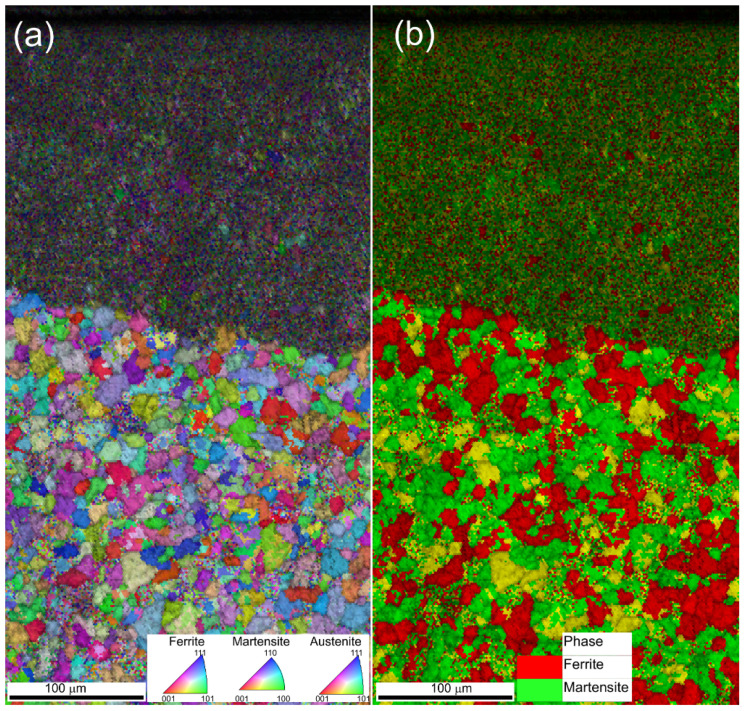
EBSD maps of a disk made of X38CrMoV5-1 tool steel without electro-machined surface for (**a**) raw material and (**b**) after LP treatment.

The EBSD maps are also shown in Figure 13b,c. Figure 13b shows the inverse pole figure (IPF) of the surface and reveals the grains. The re-melted layer consists of relatively large grains that grow through the complete layer thickness (columnar growth) situated at the top right of Figure 13b,c. The HAZ and bulk microstructures are not distinguished by these measurements. The main difference is an increase in black regions (grain boundaries) which is a sign of worse quality of the EBSD map. This is due to a martensitic transformation that creates a large strain of the lattice and small grains. Figure 13c shows a phase distribution of austenite, martensite and ferrite. It is surprising that given the high cooling rate observed in LP, the re-melted layer consists only of residual austenite. An explanation is that the electro-machined surface has a different carbon concentration as compared to the bulk material (see Table 8). The reason is that it is known that carbon promotes austenite stability. Hence, the austenite in the re-melted layer has become a stable phase, which is not the case for the bulk of the original tool steel. To confirm this hypothesis, the same LP treatment was performed on the disk of the same material but without an EDM surface. In this sample, no residual austenite was found in the re-melted layer and the martensitic transformation occurred as evident from Figure 14. Actually, the microstructure of the re-melted layer is identical to the HAZ. The bulk microstructure was, in this case, not hardened steel and the grains are well visible outside the HAZ.

**Table 7:** Measurements of the re-melted layer and HAZ for the optimization tests.

Tests Coded Factors	DoE Parameters Real Values				Power	Responses			
	*Ø*	*v*	*T*	*O*		Width Melt	Depth Melt	Width HAZ	Depth HAZ
	[mm]	[mm/s]	[°C]	[%]	[W]	[µm]	[µm]	[µm]	[µm]
Optim 1 (1,−1,−1,1)	0.9	20	1940	-	326	880	40	1133	262
Optim 2 (1,−1,0,1)	0.9	20	2080	-	359	964	60	1230	291
Optim 3 (1,−1,1,1)	0.9	20	2220	-	391	1015	87	1238	322
Optim 4 (1,0,−1,1)	0.9	35	1940	-	365	809	31	1046	213
Optim 5 (1,0, 0,1)	0.9	35	2080	-	401	850	36	1047	223
Optim 6 (1,0,1,1)	0.9	35	2220	-	438	931	43	1086	254
Optim 7 (1,1,−1,1) = DoE 12	0.9	50	1940	-	399	629	19	896	142
Optim 8 (1,1,0,1)	0.9	50	2080	-	438	821	28	998	200
Optim 9 (1,1,1,1)	0.9	50	2220	-	478	871	42	1050	211

**Table 8:** Chemical composition measured on the EDM surface and on for the bulk, below is the target values defines for X38CrMoV5-1 steel.

	C	Si	Mn	P	S	Cr	Mo	Ni	Cu	V	W	Fe
	%	%	%	%	%	%	%	%	%	%	%	%
EDM	1.68	0.901	0.273	0.016	0.003	4.847	1.274	0.206	1.44	0.466	0.128	88.63
Bulk	0.417	0.849	0.333	0.009	0.003	5.007	1.253	0.227	0.066	0.444	0.105	91.15
Min.	0.33	0.8	0.25			4.8	1.1			0.3		
Max.	0.41	1.2	0.5	0.03	0.02	5.5	1.5			0.5		

## 4. Conclusions

Laser macro polishing (LP) experiments, using a continuous wave (CW), high-power diode laser with a 980 nm wavelength of an X38CrMoV5-1 (DIN 1.2343) tool steel with rough EDM surface have been reported. A DoE approach was employed to find the optimum process parameters for industrial applications. The four selected factors were the spot size (*Ø*), the velocity (*ν*), the maximum temperature (*T*) and the overlap (*O*) between two successive parallel lines. The uniqueness of this contribution is by selecting a numerically simulated maximum temperature of the melt pool as the power-related parameters instead of the laser power itself. A first DoE was performed to find the significant parameters and the region of the optimum. It was found from Equation (3) that, in decreasing importance, the velocity (*v* = +0.25), temperature (*T* = −0.20), and overlap (*O* = −0.14) are the main parameters influencing the line roughness *Ra* (defined as surface variations with a wavelength below 0.8 mm). With the model developed, the direction of the optimum is found by increasing the temperature from 1940 to 2220 °C, and reducing the minimum velocity from 50 to 20 mm/s. The processing time is also an important parameter for industry viability. Hence, to keep this process competitive, it was decided to keep the overlap at the maximum value of 90%.

The first DoE reveals also that the influence of the process on the waviness *Wa* (wavelength above 0.8 mm) is not as strong as the critical wavelength and evidence of this is in Equation (4). This is due to the fact that LP can process is limited by the melt pool size. The model for the waviness is not as precise (*R^2^* = 0.91 for *Ra* and 0.66 for *Wa*) and contains the temperature (*T* = −0.091), the spot size (*Ø* = −0.054) and two interactions. The first interaction is between the velocity and temperature (*vT* = +0.09) and the second is between the spot size, temperature and overlap (*Ø TO* = −0.077). The optimum is, similarly to the roughness model, found in the direction of higher temperatures and overlaps and lower velocities. The only difference with the roughness model is the spot size; a bigger spot size leads to further reduction in the waviness. Thus, the optimization was performed with the larger spot size.

The optimized DoE showed that the optimum conditions for the roughness cover a wide range of conditions from 20–50 mm/s with a temperature range between 2080 and 2220 °C with a spot size of 0.9 mm and an overlap of 90%. In these conditions, the final roughness is between 0.5 and 0.55 µm. 

The optimal process parameters for the waviness cover a smaller region as there is a clear optimum for the temperature at 2080 °C with a velocity between 35 and 50 mm/s, a spot size of 0.9 mm and an overlap of 90%. Under these conditions, the final waviness is around 0.9 µm. Any further increase in the temperature leads to a slight increase in waviness and this could be due to the transition from shallow surface melting to surface over melting. 

We also found that additional reduction in the roughness is not possible with just one scan of the surface and further reductions have to be made with a second step of LP or another polishing method. 

Finally, the microstructure of single lines of LP on EDM surfaces showed that the re-melted layer is 100% of residual austenite. This can be explained by the uptake of austenite stabilizing carbon at the surface of the EDM surface. On similar tests performed on non-EDM surfaces, the austenite was as expected for this alloy and the high cooling rate transformed into martensite.

## Data Availability

Not applicable.
